# Dynamic integration of biological data sources using the data concierge

**DOI:** 10.1186/2047-2501-1-7

**Published:** 2013-02-04

**Authors:** Peng Gong

**Affiliations:** 1 Biomedical and Multimedia Information Technology (BMIT) Research Group, School of Information Technologies, the University of Sydney, Sydney, NSW 2006 Australia; 2 Department of PET and Nuclear Medicine, RPA Hospital, Camperdown, NSW 2050 Australia

**Keywords:** Biology, Middleware, Data integration, Ontology, State machine

## Abstract

**Background:**

The ever-changing landscape of large-scale network environments and innovative biology technologies require dynamic mechanisms to rapidly integrate previously unknown bioinformatics sources at runtime. However, existing integration technologies lack sufficient flexibility to adapt to these changes, because the techniques used for integration are static, and sensitive to new or changing bioinformatics source implementations and evolutionary biologist requirements.

**Methods:**

To address this challenge, in this paper we propose a new semantics-based adaptive middleware, the Data Concierge, which is able to dynamically integrate heterogeneous biological data sources without the need for wrappers. Along with the architecture necessary to facilitate dynamic integration, API description mechanism is proposed to dynamically classify, recognize, locate, and invoke newly added biological data source functionalities. Based on the unified semantic metadata, XML-based state machines are able to provide flexible configurations to execute biologist's abstract and complex operations.

**Results and discussion:**

Experimental results demonstrate that for obtaining dynamic features, the Data Concierge sacrifices reasonable performance on reasoning knowledge models and dynamically doing data source API invocations. The overall costs to integrate new biological data sources are significantly lower when using the Data Concierge.

**Conclusions:**

The Data Concierge facilitates the rapid integration of new biological data sources in existing applications with no repetitive software development required, and hence, this mechanism would provide a cost-effective solution to the labor-intensive software engineering tasks.

## Background

High throughput experimental processes in life science have led to a large variety of biological data sources continuously emerging on the Internet [1, 2]. These data sources provide great research potential for biology researchers to obtain data that support their new biological insights in areas such as gene prediction, proteomics analysis, mutations, and drug discovery. However, biology information is not easily and conveniently accessible [3, 4]. Even though most biological data source suppliers provide tools to access their own data sources, biologists have to switch to different interactive interfaces and manually seek and combine results from different resources. This manner of information collection is consequently tedious and time consuming [5, 6]. Hence a unified access mechanism to these various biological data sources has been necessitated to improve biology research processes [7].

Many practical decisions have led to heterogeneous implementations of the existing biological data sources. Regardless, the resulting complexity makes the integration of biological data sources difficult. The lack of standardization also means biological data is available in a wide variety of formats. Various data schemas such as flat files, structured data (e.g. database), semi-structured data (e.g. XML [8]), and arbitrary data structures, result in syntactical difficulties for data unification [9]. Multifarious data access mechanisms such as web page navigation, web services, remote database access, FTP, Email, Wiki [10], and so on, pose technical obstacles to unified schemes for data extraction and communication. In addition, semantic problems arise due to no standard terminology conventions in biological data. For instance, integration conflicts arise when different notions use the same terminology, or the same concept has different representations in different sources.

Data source integration is consequently a challenging research topic in biological data, and large amount of research efforts have been devoted into this area [11]. Early approaches focused on the integration of multiple biological relational databases. For instance, one popular early integration approach used multi-database query languages such as the Collection Programming Language (CPL) [12], to enable biologists to specify complex queries for different biological databases. Mediation systems, for example Mediator-Wrapper [13], Database Federation [14], and data warehousing [15], are another trend to provide a virtual or physical view of global biological data schema. In addition, some biological data centers such as EBI [16], NCBI [17], and DDBJ [18], use navigation oriented methods, such as web browsers, to provide customized queries for researchers to access linked data sources. More recently, structured integration has gradually evolved to semantics based integration [19] and Web service based integration [20]. In these approaches, XML based Web services use Internet standards and protocols such as UDDI, SOAP, and WSDL, to offer interoperable and expandable integrations of biological data sources. Finally, targeting semantic heterogeneity in biological data sources, ontology-driven data integration [21, 22] has developed standardized biological vocabularies and naming convention [23].

Although these existing technologies can partially solve the basic integration problems of the distribution, heterogeneity, and autonomy of biological data sources, they lack sufficient flexibility in adapting to the inherently dynamic and evolutionary environments. New research challenges emerge with the increase in scale and diversity of new biological data sources.

First of all, with the constant advance in bioinformatics techniques, for instance, microarray, new types of biology data with heterogeneous formats are continuously created and developed from different data sources. It is challenging to integrate new data schemas of these data types into existing integration systems at runtime. Furthermore, along with new types of biology data, new bioinformatics services for querying, translation, analysis, computation, and visualization, are continuously appearing for biologists to exploit their bioinformatics research processes. The collaboration of all these services in a uniform and automatic manner would greatly benefit bioinformatics research performance. However, in conventional integration approaches, these bioinformatics services are machine-manipulated but not machine-understandable. Once their invocation protocols have been hard-coded into the integration system, they cannot be simply modified if the logic sequence of operations required is updated. Adding new services or any changes of access interfaces would inevitably increase the burden of software development and maintenance.

Traditional integration techniques cannot solve the above dynamic challenges, because their static coding is sensitive to changes of biological data source functionality and schema. An integrated system has to be kept up-to-date by manually modifying programs when new biological data sources must be integrated or the features of integrated biological data sources must be changed. The required software engineering tasks are time consuming, error prone, and expensive, and the inertia they introduce cannot accommodate to the growth of biological data sources on the Web [24]. Therefore, the next generation of integration technologies for bioinformatics should have the ability to rapidly respond to changing requirements and dynamic environments.

To address the above issues, in this paper we demonstrate how the Data Concierge adaptive middleware platform [25, 26] can be extended to integrate new biological data sources without the need for application-level programming. Our approach provides a comprehensive solution that can be used to dynamically connect to, access, and manipulate multiple biological data sources from a single client interface. The uniform access mechanism allows biologists to easily perform advanced and efficient research tasks in dynamic data environments.

## Methods

### Data concierge architecture

**Figure 1:**
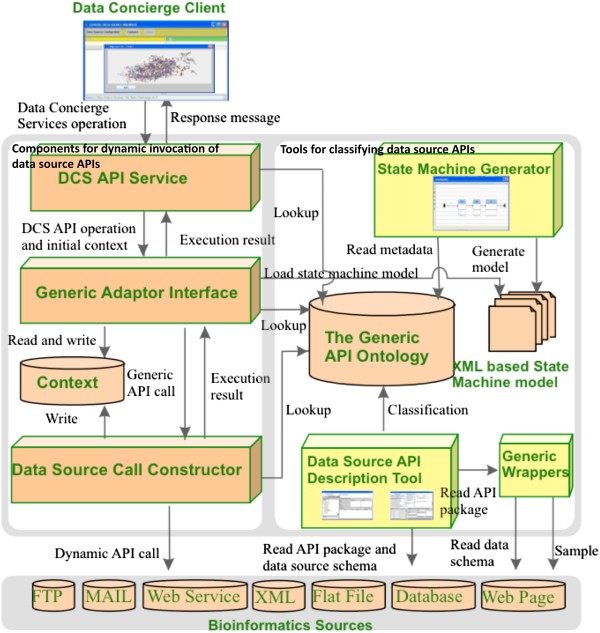
**The architecture of the data concierge.**

The Data Concierge has been proposed to address the above mentioned challenges in the dynamic integration of biological data sources. The architecture of the Data Concierge is portrayed in Figure 1, which has sufficient flexibility to provide a suitable infrastructure for dynamic and evolutionary bioinformatics environments. It utilizes reflection and knowledge representation to support introspection and adaptation to the available biological data source collection.

The architecture depicts two distinct subsystems, namely tools for classifying data source APIs, and components for dynamic invocation of data source APIs. The tools for classifying data source APIs are used by software engineers to describe the necessary metadata for bioinformatics source API. The tools are *Generic Wrappers*, *Data Source API Description Tool* and *State Machine Generator*. The components for dynamic invocation of data source APIs consists of *DCS API Service*, *Generic Adaptor Interface* and *Data Source Call Constructor*.

#### Tools for classifying data source APIs

##### Generic wrappers

Data Concierge has the capabilities to classify data source API into Generic API ontology and to generate state machine models for user-level data source operations. However, for some data sources such as Web pages and plain text, there are no specific APIs for Data Concierge to classify into Generic API Ontology. Therefore, generic wrappers are provided for the API classifications.

The Data Concierge creates generic wrappers for some specific types of biological data sources, such as text, XML, Web page, and database. They can ease the Data Concierge’s manipulations on these biological data sources, and reduce the complexities of constructing the related state machine models. These generic wrappers in Data Concierge include XML wrapper, text wrapper, Web page wrapper, and relational database wrapper.

**Figure 2:**
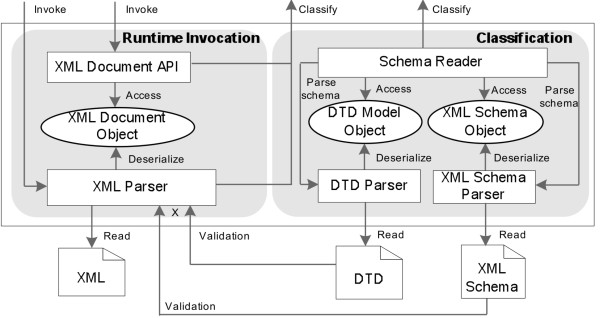
**The architecture of generic XML wrapper.**

*Generic XML Wrapper* provides the capabilities of parsing and exacting data from customized XML documents, as illustrated in Figure 2.

1. When a new XML document needs to be integrated into the Data Concierge, its schema file, DTD or XML Schema format, first is parsed and deserialized into the corresponding internal schema model objects.
2. Then, from the generated internal model objects, *Schema Reader* component extracts all XML elements and attributes which are described in schema file.
3. Finally, the extracted elements and attributes are classified into the generic API ontology. The query entries of these elements are represented in the format of paths from the top root node to the corresponding described nodes.

At runtime invocation, when Data Concierge accesses to an instance of integrated types of XML documents, Data Concierge invokes the classified API methods from *XML parser* components for parsing and deserializing XML documents into an internal XML document object. Then it delivers the classified element or attribute paths to *XML Document API* component and extracts required data from the internal XML document instance.

**Figure 3:**
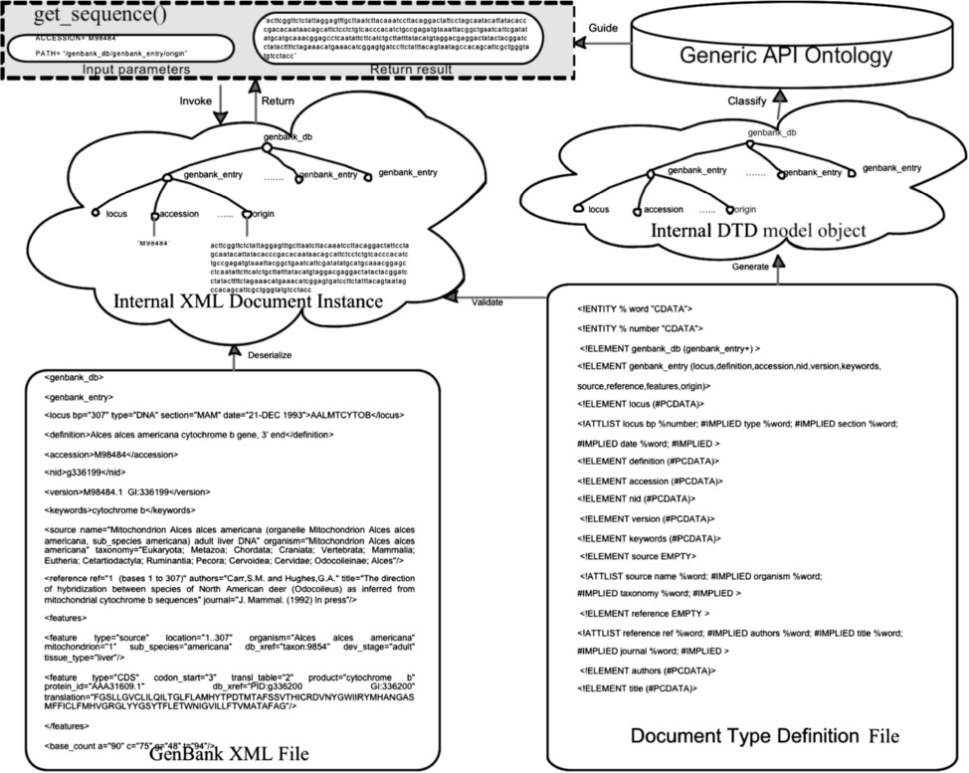
**An example of XML file integration.**

Figure 3 gives one example of the integration of GenBank XML files. As illustrated in this diagram, DTD elements such as “*genbank_db*”, “*Genbank*_*entry*”, “*accession*”, and “*origin*”, are presented as tree nodes of an internal DTD model object. To locate data in internal XML document instances, for example, reading a DNA sequence form an XML file, Generic XML Wrapper classifies corresponding tree paths, such as “*/genbank_db/genbank_entry/origin*”, into the Generic API Ontology.

Similar to *FlatEx*[27], the component of *Generic Text Wrapper* is designed to automatically extract biological data from various biological structured flat files.

**Figure 4:**
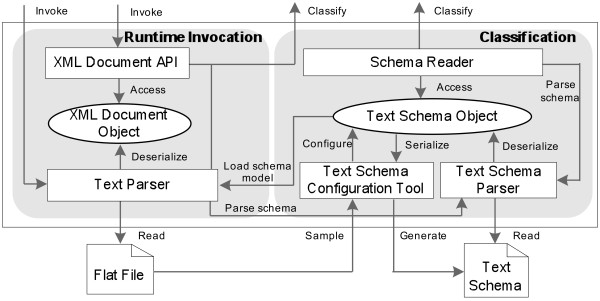
**The architecture of generic text wrapper.**

As illustrated in Figure 4, to be integrated into the Data Concierge, a new type of structured flat file will be first sampled for generating its text schema file. The *Text Schema Configuration Tool* defines *Tokens*, delimiting patterns, and other Meta elements according to sampled text documents. All extracted metadata are configured and recorded into an internal *Text Schema Object*. Then the generated *Text Schema Object* instance is parsed and classified into the Generic API ontology, and is serialized into corresponding text schema file.

At runtime, to access an instance of the integrated flat file type, the configured text schema file is deserialized into the corresponding internal text schema object, which guides the Text Parser component to parse the structured flat file instance into specific XML document instance. The internal XML document instance is in a tree structure. The elements of the parsed XML document objects are obtained by using XPath-like access mechanism which is similar to The *Generic XML Wrapper*.

In *Generic Text Wrapper*, the internal *Text Schema* object is critical to data extraction from customized flat files. It defines delimiting patterns for every text node and describes the hierarchical structure of text documents. The *Text Schema* guides the *Generic Text Wrapper* to parse corresponding structured flat files into internal XML document instance.

**Figure 5:**
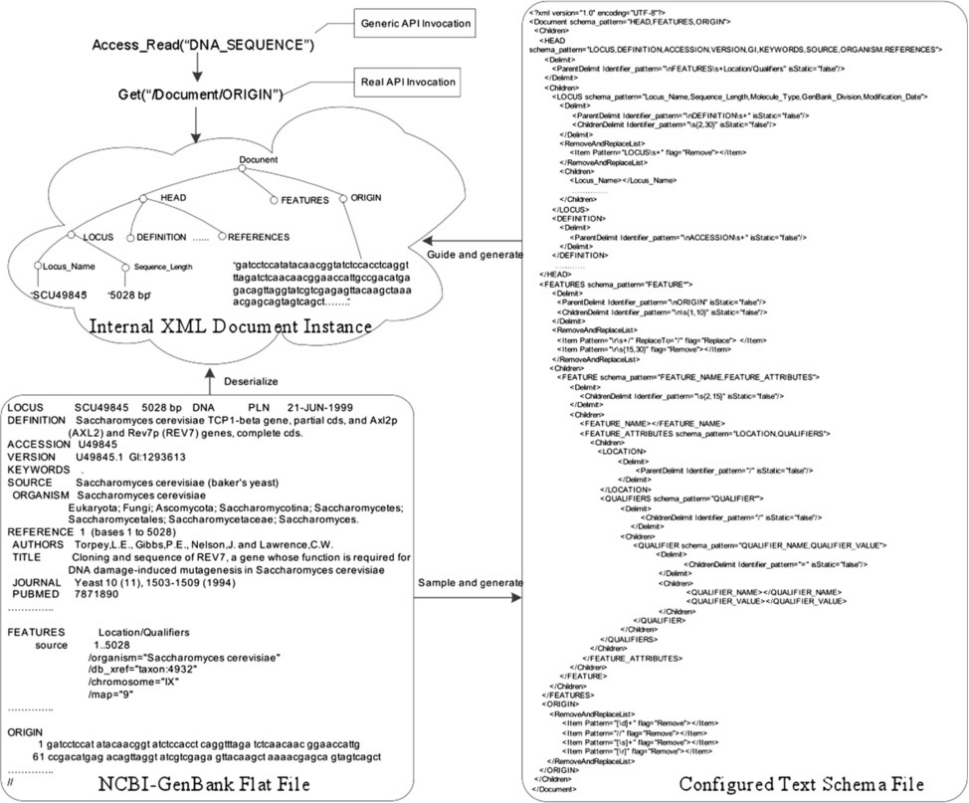
**An example of structured flat file integration.**

An example for the integration and runtime access of NCBI-GenBank flat file type is illustrated in Figure 5. The NCBI-GenBank flat file instances are sampled for generating text schema file. Some unchanged terms such as “*LOCUS*”, “*FEATURES*”, and “*ORIGIN*” are recognized as tokens. Delimit patterns are formed by the composition of tokens and regular delimiters such as Space, Return, and punctuations. For example, the delimit pattern of “*\nFEATURES\s+Location/Qualifiers*” is used to separate the *HEAD TextNode* with others.

**Figure 6:**
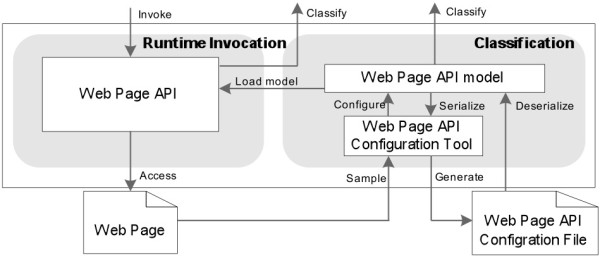
**The architecture of generic web page wrapper.**

To integrate various biological Web pages, *Generic Web Page Wrapper* (Figure 6) is designed, which contains components *of Web Page API Configuration Tool*, *Web Page API model*, and *Web Page API*.

When a new Web page needs to be integrated into the Data Concierge, the *Web Page API Configuration Tool* monitors user’s Web page interaction processes and extracts metadata from the sampled Web pages, corresponding HTTP requests, and HTTP responses. The obtained metadata are configured into an internal *Web Page API model*, which is finally serialized into a Web page API configuration file.

*Web Page API* model includes necessary information to issue HTTP requests and filter results from HTTP responses and helps the Data Concierge to automatically interact with Web pages and extract user-interested data at runtime.

**Figure 7:**
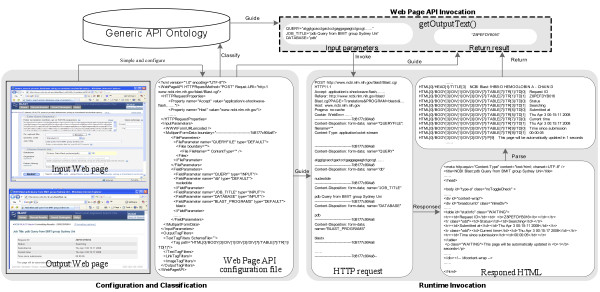
**An example of web page integration.**

Figure 7 illustrates the procedure of integrating an NCBI-Blast Web page. When this Web page needs to be integrated into the Data Concierge, it first needs to be sampled and configured into *Web page API configuration file*.

In the Web page API configuration file, Web page URL and HTTP request method “*POST*” are configured as attributes of WebPageAPI element. HTTP request properties such as “*Accept*” and “*Host*”, are sampled from real HTTP interactions and default values of these properties are saved into this configuration file. User input parameters such as “*QUERY*”, “*JOB_TITLE*”, and “*DATABASE*”, and other default parameters such as “*QUERYFILE*”, “*db*”, and “*BLAST_PROGRAMS*”, are configured as multipart-form data contents with an appropriate boundary. In addition, a text tag filter with the path of “*HTML[0]/BODY*[3]*/DIV*[1]*/DIV*[3]*/DIV*[7]*/TABLE*[7]*/TR*[1]*/TD*[1]” is set to extract job ID from the responded Web page. These metadata are classified into the *Generic API Ontology*.

At runtime, when the Data Concierge accesses data from the integrated Web page, the configured metadata of the *Web page API configuration file* guide *Generic Web Page Wrapper* to generate correct HTTP requests. Then *Generic Web Page Wrapper* parses the responded Web page into a serial of tag paths. According to the text tag filter which is configured in Web page API configuration file, the job ID “*Z8PEFDYB016*” is extracted according to the provided tag paths.

**Figure 8:**
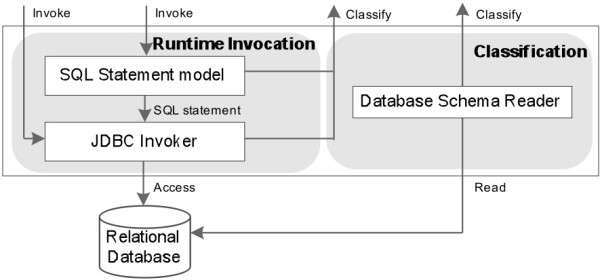
**The architecture of generic relational database wrapper.**

*Generic Relational Database Wrapper* (Figure 8) is designed to integrate diverse relational databases into the Data Concierge. This wrapper contains components of *Database Schema Reader*, *SQL statement model*, and *JDBC invoker*.

When a new relational database needs to be integrated, the *Database Schema Reader* extracts tables and field columns from the database, and classifies these metadata into the Generic API Ontology.

*SQL statement model* defines basic elements for creating various SQL statements such as *Insert*, *Select*, *Update*, and *Delete* and is used to build SQL statements for the automatic access to integrated databases. To access the relational database at runtime, the Data Concierge uses classified API methods to equip an instance of *SQL statement model* with values from state machine context. After deparsing this instance into the corresponding SQL statement, The *JDBC Invoker* uses the deparsed SQL statement to manipulate the integrated relational database.

**Figure 9:**
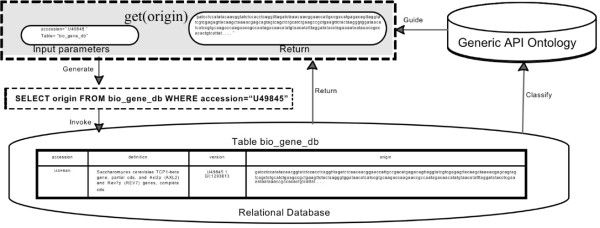
**An example of relational database integration.**

As illustrated in Figure 9, the schema of table *bio_gene_db* is firstly classified into the Generic API ontology. According to the classified results and input parameters, *Generic Relational Database Wrapper* selects appropriate SQL pattern and dynamically constructs a Select SQL statement, which is further invoked by *JDBC Invoker*. Its final JDBC execution result is the required value of the field *origin*.

##### Data source API description tool

The *Data Source API Description Tool* facilitates the construction and maintenance of a Generic API Ontology. It uses Java Reflection to access biological data source API, and then the tool user classifies the API as metadata described in the Generic API Ontology. Using this tool, biological data source schemas and their corresponding marshaling methods are categorized into unified terminologies in the ontology.

##### State machine generator

The *State Machine Generator* is a graphical description tool, which facilitates the generation of XML-based state machine models that describe a sequence of Generic API calls. The state machines provide flexible configurations for complex API access protocols with features of quick changes or multiple usages.

#### Components for dynamic invocation of data source APIs

##### DCS API service

The *DCS API Service* implements the Data Concierge Services (DCS) API as a set of Web services. Each DCS API specifies an abstract operation that can be performed on biological data sources. The *DCS API Service* processes biologist’s request by searching the ontology for the operations.

##### Generic adaptor interface

The *Generic Adaptor Interface* parses and executes DCS API operations. Simple DCS API operations map to a generic API call for the data source, while complex operations reference a state machine that generates a sequence of generic API calls.

##### Data source call constructor

The *Data Source Call Constructor* translates generic API operations to specific data source API calls through the mappings defined in the Generic API Ontology. Reflection is used to dynamically construct API calls to data sources.

### Integration of new bioinformatics sources

In this section, we give an illustration on how the Data Concierge is used to integrate new biological data sources at runtime.

#### Construct and maintain generic API ontology

The dynamic feature of efficient access to new types of biological data sources in the Data Concierge is achieved by the Generic API Ontology. In Data Concierge, such knowledge-based integration model is used to sample and classify the contents of biological data sources and their functionalities, and to collect the biologists’ data interests and preferences. For a clear illustration, we separate the Generic API Ontology into two parts with different usages. One is used for the dynamic construction of data source API calls. The other is the generic API ontology for improving the flexibility of client applications.

**Figure 10:**
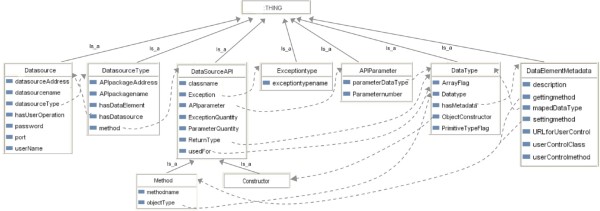
**The generic API ontology for the semantic description of data source API.**

The challenges caused by unpredictable changes could be tackled if the integration system were able to dynamically connect and invoke previously unknown APIs on the basis of the semantics of source functionalities. The top-level model of the first part in the ontology (Figure 10) is used by the Data Concierge to dynamically construct calls to data sources APIs. It represents the semantics of the data source API and associated data schema. The classified metadata in the Generic API ontology shield the heterogeneities of low-level source interfaces and data models from the Data Concierge middleware and client applications. After classifying data source APIs and data schemas into the Generic API Ontology with the *Data Source API Description Tool*, these metadata help the Data Concierge to discover and invoke the desired biological data source functionalities for accomplishing biologist manipulations.

**Figure 11:**
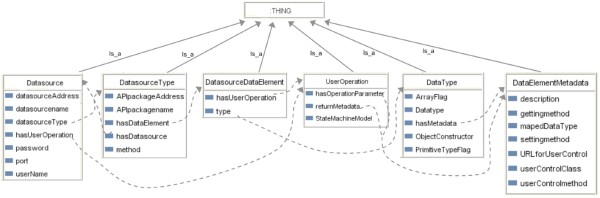
**The generic API ontology for client application usages.**

To be able to access biological data sources dynamically, client applications and user GUIs must be able to discover newly available biology data types and functions, and utilize the results meaningfully. To make it possible for a user GUI to be created that can display data from data sources with a priori unknown content, semantic information of query results must be available. Figure 11 illustrates the ontology structure for this purpose. In this structure model, the classified abstract metadata, which takes charge of interpreting biologist manipulations, provide flexible mechanisms for biologists and client applications to dynamically and transparently access new integrated biological data sources.

The Generic API Ontology mechanism and configurable state machine models endow the Data Concierge and client applications with the capability of flexible adaptation to the changes of biological data sources.

**Figure 12:**
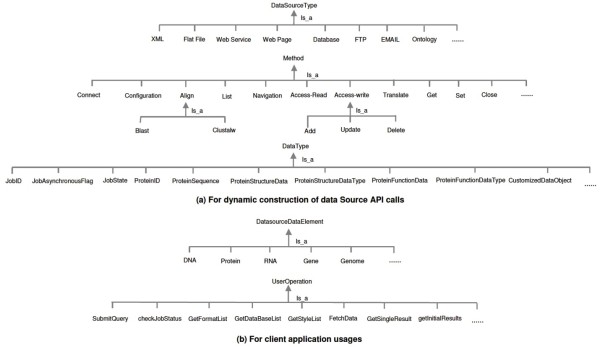
**The extension of the generic API ontology for biological domain.** (a) For dynamic construction of data source API calls. (b) For client application usages.

As illustrated in Figure 12, the Generic API Ontology dynamically extends with the increase of new types of integrated biological data sources. The *DataSourceType* Class covers but not limits to source types such as XML, flat file, web page, web service, relational database, FTP, email, and ontology. Some biological analysis and computing functions such as *Blast* and *Clustalw* are classified into the *Method* Class. Subclasses of the *DataType* Class provide unified terms for both biological domain and computer computations. Biologists utilize these metadata to customize their specific user operations such as *submitQuery* and *FetchData*, on their interested *DatasourceDataElements* such as *Gene*, *Protein*, and *DNA*.

#### Classify biological data source API and generate configurable state machine models

When a new biological data source needs to be integrated into the Data Concierge, its functionalities and accessible biological data types have to be classified into the Generic API Ontology by the *Data Source API Description Tool*. The *Data Source API Description Tool* uses Java reflection to present the API method signatures and parameter types to a data source API expert for classification.

**Figure 13:**
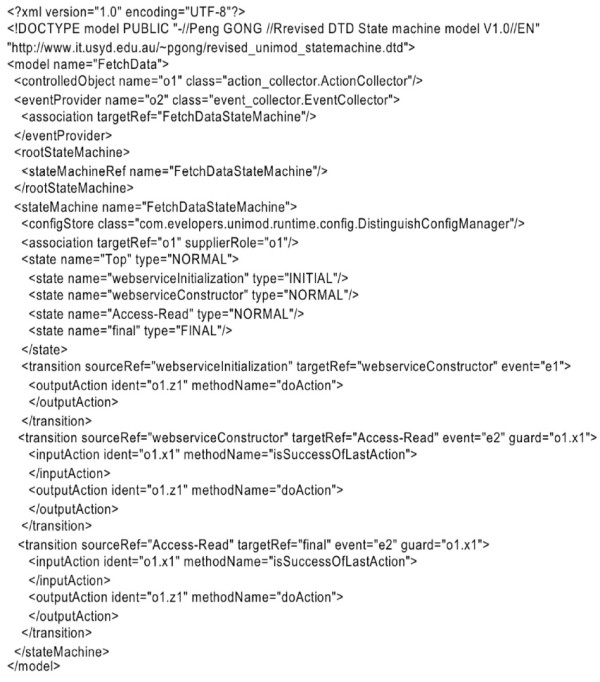
**An example of state machine model.**

In order to support dynamic data exchange, customized data schemas in biological data sources need to be decomposed into data elements that the integration system can recognize and use. The execution of this dynamic transformation depends on the knowledge in the Generic API Ontology. After the classification of biological data source API, the *State Machine Model Generator* is used to generate XML-based state machine models. Biologists can customize their own state machine models for their specific interests by using a downloaded *State Machine Generator*. The XML-based state machine models provide flexible configuration for various complex operations relevant to biologists, which requires a sequence of biological data data source API functions. The Data Concierge interprets the state machine models at run time to dynamically construct calls to each data source API in the sequence. The XML-based state machine models are based on Unimod [28] using SWITCH-technology [29], and follow Event-Condition-Action (ECA) rules, which take the form of ON Event IF Condition DO Action, to express control flows in state machines. These rules specify event trigger and guard conditions for each action. An action is executed when the triggering event occurs, if and only if the guard condition is true. In the following example of an XML-based state machine model, (Figure 13) three Generic API operations, webserviceInitialization, *webserviceConstructor*, and *Access-Read* are sequentially executed for biologist’s *FetchData* operation.

#### Invoke biological data source operations at run time

**Figure 14:**
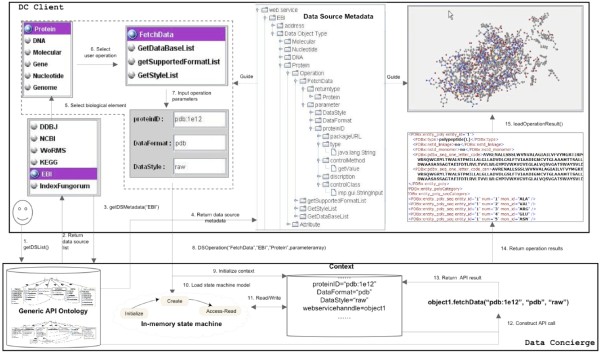
**Fetching protein data using data concierge.**

New biological data sources and related user operations are available to DC Clients after classifications. Biologists can operate new integrated biological data sources at run time. For example, the sequence in Figure 14 illustrates how a biologist uses the Data Concierge to access a new integrated EBI biological Web service [30] and to perform *FetchData* operation on *Protein* data.

**Figure 15:**
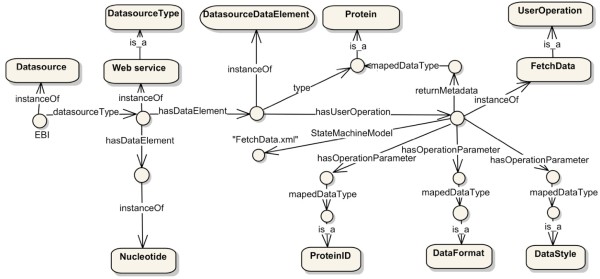
**An example of classified results for biological client applications.**

Through invoking *getDSList()* (step 1), DC client gets integrated biological sources from Data Concierge (step 2). Then the list guides a biologist to select a data source of interest, e.g. *EBI*. After the selection, DC client will issue *getDSMetadata(EBI)* method (step 3), which is a Web service and aims to extract the data source metadata classified in the Generic API Ontology. As results, the metadata extracted from Figure 15 contribute to form data source tree view illustrated in Figure 14 (step 4).

The returned metadata guide the biologist to do the *FetchData* operation on *Protein* and guide the DC client to load appropriate components for preparing parameters of the *FetchData* operation (step 5–7). For example, the *ProteinID* can be obtained through the invocation of *getValue* method in *imp.ui.StringInput* class.

After preparing parameters, DC client sends the *FetchData* operation request to Data concierge by calling an available DCS API with the format of *DSOperation(“FetchData”, “EBI”, “Protein”, {parameter array})* (step 8).

Upon receiving the client request, Data Concierge initializes context (step 9) and loads configured state machine model (step 10), *FetchData.xml*, for the *FetchData* operation. The state machine model is executed by a state machine engine which issues a sequence of Generic API calls such as *Initialize*, *Create*, and *Access-read* (step 11–13).

**Figure 16:**
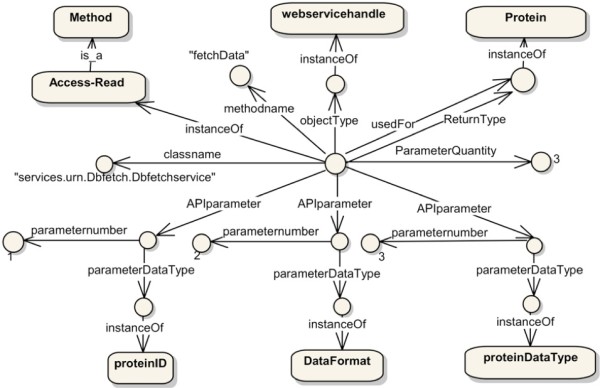
**An example of classifying biological data source functionality.**

During the execution of the loaded state machine, every generic API will be mapped to corresponding data source API. For example, following the ontological definition for the *fetchData* API (Figure 16) and the state machine context, *Access-read* is translated to *object1.fetchData(“pdb:1e12”, “pdb”, “raw”)* (step 12).

Finally, the execution result of the *FetchData* operation is sent to the client application (step 14). The extracted metadata guides the DC client to load appropriate class and invoke its method for displaying obtained protein data (step 15).

## Results and discussion

### Performance

We have implemented the Data Concierge dynamic integration mechanisms with FTP, SMTP, POP3, and some biological data sources.

For the integration of biological data sources at runtime, Data Concierge sacrifices extra performance on reasoning knowledge models and dynamically doing data source API invocations. To investigate the overheads of using Data Concierge, some validation experiments were carried out. The experiments on the middleware part were performed on 2 machines connected by a local area network (100M bps):

1. a 2×2.4 GHz Mac Pro with 16 GB RAM and Mac OS X Version 10.6.7, executing the *Data Concierge Web Service* layer;
2. a 3.00 GHz PC with 2 GB of RAM and Windows XP Service Pack 2, executing the *Generic Adapter Interface* and *Data Source Call Constructor* components.

#### Tests on generic API ontology

**Figure 17:**
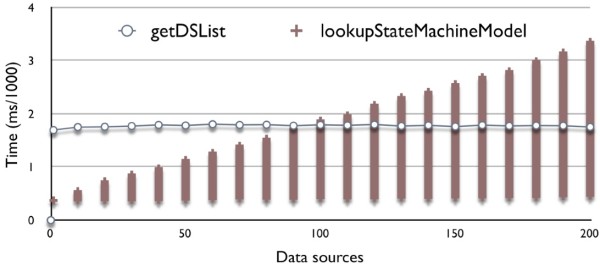
**Tests on getDSlist and lookupStateMachineModel.**

As illustrated in Figure 17, the performance of *getDSList* operation remains stable with the increase of integrated data source instances (up to 200), which costs about 0.0018 ms to get data source list from the Generic API ontology. However, because we take iterative comparing and matching state machine models in the algorithm of *lookupStateMachineModel*, the results on locating corresponding state machine models are different. The minimum performance value remains at about 0.00037 ms while its maximum increases linearly with integrating new data sources.

**Figure 18:**
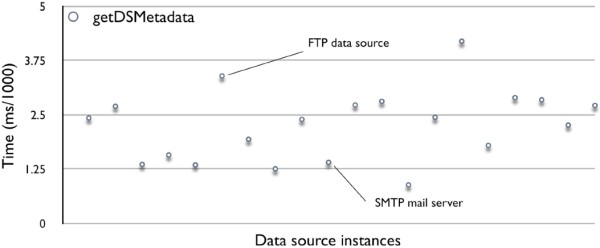
**Tests on getDSMetadata.**

In Figure 18, the performance of *getDSMetadata* varies for different data sources. It is decided by how many meta attributes in Generic API Ontology are associated with each data source instance. If one classified data source instance has more associated meta attributes such as data elements and related user operations than the others, Data Concierge will spend a much longer time on querying these metadata from Generic API Ontology. For example, the performance of getting metadata of an FTP data source, which includes data elements *File* and *Directory* and their user operations (such as *Read*, *Write*, *Delete*, *Up_Navigation*, *Down_Navigation*) and other attributes (such as *name*, *size*, *date*, *type*, *userID*, *groupID*, *permissions*, *numberofLinks*, etc.) is 0.0034 ms. While querying metadata for a simple SMTP mail server that has *Mail* element, *Write* operation, and some simple attributes, is only 0.0014 ms.

#### Tests on null methods

The real performance and functionality of the Data Concierge are related to classified data source functions and their collaborations. To avoid interference from the internal implementation of biological data source functions, null methods are used to evaluate the performance of our Data Concierge.

To compare generic API invocation with the corresponding simple static API call, we created a null method *m()*, which does not have any parameter in its API definition. In the first row of Table 1, the average overhead of the static method call on this null method is 0.000042 ms. While the overhead of the generic API call on this null method is 0.116487 ms, in which the binding time from generic API call to real data source API call costs 0.08748 ms and java reflection invocation costs about 0.029 ms.

**Table 1:** **The performance results of generic API invocations and static method calls on different null methods**

Null method	Generic API call (1000 calls/1000 ms)			Static method calls (1000 calls/1000 ms)
	Binding Time	Invocation time	Total	
No parameter()	0.087480	0.029007	0.116487	0.000042
One parameter m(C1 p1)	0.278896	0.027818	0.306714	0.000974
Two parameters m(C1 p1, C2 p2)	0.465648	0.028491	0.494139	0.001772
Three parameters m(C1 p1, C2 p2, C3 p3)	0.713984	0.029231	0.743215	0.002601

If there are no required parameter types existing or available in state machine context when constructing an API method call, the performance of Generic API invocation will be affected by constructing these required API parameters from Context. Therefore, we also created three other null methods *m(C1 p1)*, *m(C1 p1, C2 p2)*, and *m(C1 p1, C2 p2, C3 p3)* which have different numbers of parameters, to test the impact of state machine context on generic API invocations and state machine models. The performance comparison results of different null methods (Table 1) show that the binding time is affected by state machine context. If there are no required API parameter types in the state machine context, the Data Concierge needs to construct corresponding parameters to issue a real data source API call after a generic API is invoked. As a result, constructing and preparing API parameters increases the overhead of generic API invocations. As shown in Table 1, constructing every parameter for a null method invocation adds about 0.2 ms to the overhead of binding time, while the java reflection invocation on real API call almost keeps the same value, 0.029ms.

**Figure 19:**
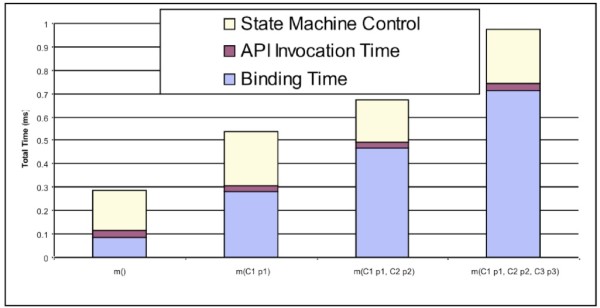
**The performance results of state machine models with different null methods.**

As shown in Figure 19, the state machine engine spends 0.15-0.25 ms on controlling and scheduling the invocation of every generic API and the overall overhead of executing state machine models increases with the API parameter complexity.

**Figure 20:**
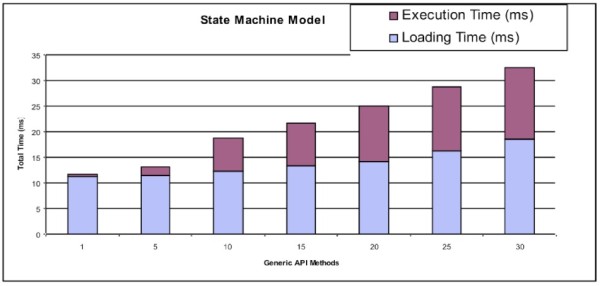
**The performance results for state machine models with different complexity.**

In addition, we tested the impact of the complexity of state machine models on the performance of the Data Concierge. We created different state machine models which have different quantities of generic API operations. All generic API operations are mapped to the same null method *m()*. As illustrated in Figure 20, the time for loading the state machine model rises slightly as the size of the state machine model increases. The more complex the state machine model, the more overhead is added to its performance.

#### Tests on biological FTP and customized data sources

**Figure 21:**
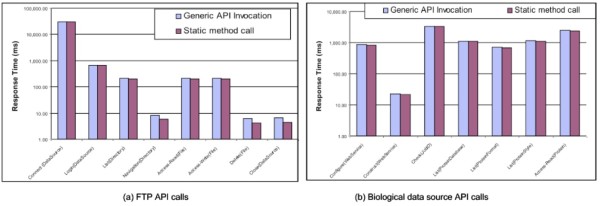
**Performance results for different generic API operations.** (a) FTP API calls. (b) Biological data source API calls.

Time spending on individual hard-coded calls directly to the methods of ftp and customized biological data source APIs was compared with the counterpart of Data Concierge generic API calls (Figure 21). Overall, querying the ontology and using Java reflection added approximately 0.2~30ms to each API call. In addition, comparison results also show that with the increase of execution time of static method calls, the extra overhead of the generic API invocations has less impact on the whole execution performance.

**Figure 22:**
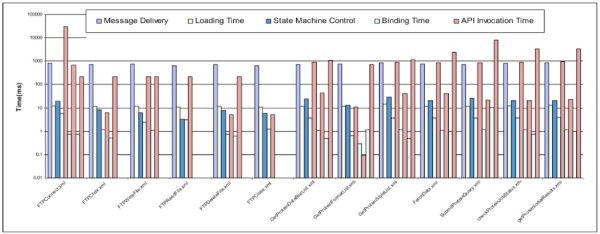
**Performance results for DCS API operations.**

Figure 22 illustrates that executing complex DCS API operations adds approximately 700~900ms overhead on their performance. This extra overhead is due to network message exchanges between *Data Concierge Web Service* and *Generic Adapter Interface* as well as loading and executing state machine models. A mechanism which can dynamically manage network communication and preload state machine models needs to be investigated in our future work, which would significantly reduce the overhead and improve the Data Concierge performance.

### Related work

Huge amounts of biological data research projects put their efforts on the data source integration issue. Some of them target at providing flexible mechanisms for various biologist requirements and quick changing biological data source environment.

*TAMBIS*[31], *BioMedoator*[32], [33], and [34] cope with frequent modification of data source schemas. Based on a domain ontology, *TAMBIS* provides homogeneous views of various data sources. The ontology is designed to relieve biologists from heavy query tasks by shielding them from low data source details and is updated to cater for schema changes in the sources. To reduce the workload of database integration, the *LIMBO* architecture in [33] is designed as light-weight approach to overcome the problems with constantly evolving data warehousing schema. Both *BioMedoator* and [34] extract and represent metadata on the content of sources and relationships among sources. *BioMedoator* aims to facilitate the easy use of this tool with no informatics training required for the biologists, while [34] targets optimizing user’s query plans according to semantic equivalence. Although these approaches provide significant flexibility to deal with user’s queries and changes of data source contents, they lack dynamic mechanisms to deal with the rapid and frequent changes of biological data source functionalities.

IBM’s *DiscoveryLink*[35] is a database middleware system that extracts data from multiple biology sources in response to a single query. This method promises there is no functionality lost in access the data source through *DiscoveryLink*. To make wrapper authoring as simple as possible, *DiscoveryLink* requires a small set of key services from a wrapper, which makes the cost of writing a basic wrapper small. Its *DiscoveryLink Server* compensates for missing functionality at data sources. This approach claims that data source schema can evolve without requiring any change in the wrapper for the reason that the wrapper does not encode information on the schema used in the data source. However, the wrapper needs to be developed when its source API is changed.

The rapid integration of biological data web pages on the Internet is addressed by the approaches of [24], [6], [36], and [37]. [24] highly depends on the description of service classes, which provides general descriptions of types of sources to integrate. However, the integrated biological data sources are restricted to biological data Web pages that must have a start page to include an HTML form with at least one text entry field. It also needs examples to analyze sources. Because of these limitations, the percentage of experimentally successful integrated web sources is lower than 70%. [6] aims to generate automatically the data source schema of each source by means of meta-information. The meta-information is extracted from output of a source query tool which identifies terms from tags of a set of Web pages examples. However, it cannot deal with some particular output formats which are provided by certain data source query tools. Based on its *OWW Search View* mechanism, [36] pursues no programming efforts for accessing any new integrated web pages. However, it cannot efficiently process user’s complex queries which include multiple joints of several data sources. [37] proposes using reconfigurable web wrapper agents for user to represent Web browsing session. Based on sequential pattern mining techniques, web wrapper agents can automatically discover and extract patterns from structurally formatted biological data Web pages. However, the initial purposes of these methods limit the scale of integrated biological data sources. These approaches will not be available to other types of data sources except Web pages.

Biological data projects such as, [38–40], and *ISYS*[41], focus on component based integrations for biological data sources. [38–40] use CORBA to dynamically integrate biological data sources. These approaches aim to achieve flexible, scalable, and reconfigurable system architecture. However, these methodologies mainly focus on specifying syntactic interfaces of integrated components, while the semantics of these interfaces are implied in their implementations. *ISYS* emphasizes a decentralized integration mechanism for dynamically synchronizing component behaviour and exchanging their services without direct knowledge of one another. However, the programming tasks of building thick client components would be heavy for the communication with others. And biologists who are not familiar with information techniques have to resort assistance from IT expertise for building their client components.

*SIBIOS*[42] and *Bio-Broker*[9] are dynamic workflow-based systems. To achieve highly adaptability, *SIBIOS* separates individual service description from wrapper engine. The service description is stored in a service schema file. This file includes domain specific knowledge described by using ontology and a set of rules that describe how the data can be extracted from the services. The wrapper engine reads these service schema files and dynamically generates specific data source wrappers. However, this approach only targets services which are provided Web pages. *Bio-Broker* is an architecture for XML-based mediator systems. The system uses *EVAS* to construct mediator-services for the integration of heterogeneous data sources. The *EVAS* benefits user to easily construct workflows for their recurrent biological data processes. However, in this architecture, wrappers are created manually.

With the emergence of Web 2.0, Mashup applications such as *Bio2RDF*[43] and *Damia*[44], provide mechanisms for users to customize new services through combining data and services from multiple Web sources. *Bio2RDF* converts various biological documents into standard RDF formats so that client applications can have unified access to various biological sources*. Damia* is a lightweight web style data integration platform which helps enterprise users to quickly combine data from different data sources and easily develop new enterprise applications. However when a new type of source appears, new wrappers such as *rdfizers* in *Bio2RDF* and connectors in *Damia* still need to be created.

The methods mentioned above provide solutions to some aspects of dynamic features in biological data source integration. The majority of these methodologies focus on the heterogeneity of data content. Some take the dynamic features of source functionalities into account. However, none of these methods uses ontology to represent the semantics of both biological data source functionality and data schema. As a result, they lack sufficient flexibility and adaptability to solve challenges arisen from dynamically integrating new previous unknown biological data sources at runtime.

### Analysis

**Figure 23:**
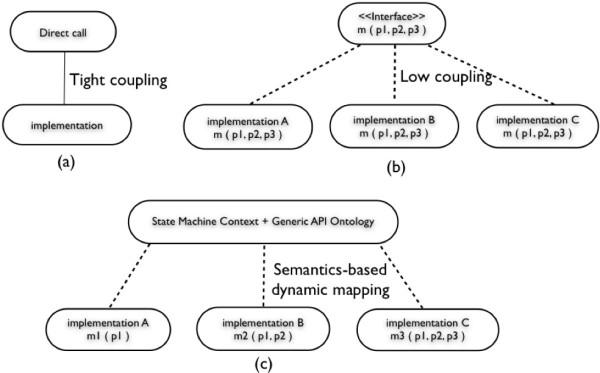
**The comparison of direct call, interface and data concierge.** (a) Direct call. (b) Interface. (c) Data concierge.

For integrating new appearing biological data sources, traditional static integration techniques (Figure 23a), which tightly couple with low level implementations, require program code changes along with subsequent testing and deployment. Although these static approaches achieve good performance, they are expensive in terms of engineering efforts.

Interface-based integration mechanisms such as CORBA and COM platforms decrease coupling with low level implementations according to their predefined Meta models (Figure 23b). They can achieve polymorphism and can dynamically compose software components and change system behaviors at run-time. However, when data source interfaces are modified, code changes cannot be avoided.

In contrast, the Data Concierge deals with interface changes without application code changes. This is because the Generic API Ontology provides a declarative mechanism that can be modified and deployed when an interface change occurs. At run-time, the Data Concierge dynamically constructs biological data source API calls based on their classified API semantics and corresponding state machine context (Figure 23c). The mechanism of dynamically accessing new data sources at run-time relieves developers from hard coded programming.

Our approach aims to use the Data Concierge to dynamically access new biological data sources at runtime without hard coded programming. This architecture provides sufficient flexibility to handle changes of biological data sources. Its dynamic call construction mechanism has several significant advantages over hard-coding calls to pre-fabricated wrappers:

- **No wrapper component is needed.** In effect, the wrapper code is constructed dynamically from the information held in the meta-data repository. This potentially significantly reduces the software engineering effort needed to connect to a new type of biological data source.
- **Ease of modification:** If the underlying data source API changes, only changes to the meta-data repository are required. As the calls to the API are dynamically constructed, the changes will take effect as soon as the meta-data is updated.
- **Hot swapping:** As there is no wrapper, updating a data source API to provide bug fixes has no downstream implications. If the interface does not change, then the Data Concierge remains oblivious to the fact that a modified version of the API has been installed.

The Data Concierge relieves programmers from the routine maintenance tasks of integrating new biological data sources. However, this approach carries the costs of building and maintaining the Generic API Ontology for a given data source, and organizing classified API metadata to build state machine models. We use the *Generic Wrappers, Data Source API Description Tool* and the *graphical State Machine Generator* to minimize the maintenance costs.

In addition, some biological data sources provide abstract functionalities. The semantics of these biological data functionalities are implied in the parameter contents of data source APIs. Therefore, it is hard for the Data Concierge to classify these data source API semantics through their API syntax. One of our future targets is to find mechanism to extract the semantics of data source functionalities from the contents of their interface parameters.

## Conclusions

To adapt to dynamic network environments and to meet diverse biologist’s requirements, we propose an adaptive middleware, the Data Concierge, to easily and rapidly integrate heterogeneous biological data sources at runtime. In this innovative architecture, the Generic API Ontology is proposed to declaratively model the semantics of data source APIs. Based on the unified semantic metadata, XML-based state machines model sequences of requests to biological data services for complex biologist manipulations. This middleware provides adaptive functionalities for both the integration system and its client application to tackle the rapid changes of biological data sources without expensive and time-consuming software development and maintenance. The costs to integrate new biological data sources in the Data Concierge are significantly lower than that of static coding integration methods.

Our future work includes enabling the Data Concierge to represent the knowledge of the biological data functionalities’ relations in the Generic API Ontology. According to classified metadata, the Data Concierge would be able to reason about the execution sequence for biologist manipulations. Thus, tasks of configuring and maintaining XML-based state machine models would be eased and even be avoided, which therefore would significantly reduce the integration costs further.
